# Current and emerging diagnostic tests available for the novel COVID-19 global pandemic

**DOI:** 10.12688/aasopenres.13059.1

**Published:** 2020-04-24

**Authors:** Gerald Mboowa

**Affiliations:** 1 The African Center of Excellence in Bioinformatics and Data-Intensive Sciences, The Infectious Disease Institute, Makerere University, Kampala, Uganda; 2 Department of Immunology and Molecular Biology, College of Health Sciences, Makerere University, Kampala, Uganda

**Keywords:** Coronavirus, COVID-19, diagnosis, genome sequencing, SARS-CoV-2, Point-of-Care test

## Abstract

On March 11, 2020 the World Health Organization (WHO) upgraded the status of the coronavirus disease 2019 (COVID-19) outbreak from epidemic to a global pandemic. This infection is caused by a novel coronavirus, SARS-CoV-2. Several rapid diagnostic tests have been developed at an astonishing pace; however, COVID-19 requires more highly specific rapid point-of-care diagnostic tests. This review describes the currently available testing approaches, as well as the available test assays including the Xpert® Xpress SARS-CoV-2 test (takes _~_45 min) and Abbott ID COVID-19 test (5 min) as easy to use point-of-care tests for diagnosis of novel COVID-19 that have so far received the US Food and Drug Administration emergency use authorizations clearance. This review is correct as of the date published and will be updated as more diagnostic tests come to light.

## Introduction

Infectious diseases are still a major threat in the world today ^1^. The diagnosis and real-time tracking of both emerging ^2^ and re-emerging infectious diseases must be reliable, fast, and affordable. Healthcare systems use different approaches to make infectious diseases diagnoses, such as clinical assessment (signs and symptoms), microscopy, microbiological cultures, radiology, molecular techniques – classical or real-time polymerase chain reaction (PCR), genome sequencing (detection presence of nucleic acid), serology (detection of the pathogens or host antibodies), artificial intelligence and machine learning ^3^. In modern medical diagnostics, whole or targeted genome sequencing or detection technologies are indispensable. In the event of novel pathogens, such as coronavirus, these technologies offer the shortest turnaround time (TAT) for results to be reported. Coupling comprehensive clinical evaluation, triaging, genomic-based diagnostics, and epidemiology to innovative digital disease detection raises the possibility of an open, global, pathogen surveillance system.

In the current case of the coronavirus global pandemic 2019, healthcare systems have the challenge of massive testing, isolating (quarantine) those suspected of being exposed to the virus, re-testing (at the end of the viral incubation period), treating and managing those who turn out to be positive, as well as real-time tracing of their contacts. The diagnosis of a novel infectious disease such as COVID-19 furthermore presents a challenge of studying and understanding its pathogenesis before developing rapid test assays to be used to subsequently diagnose cases as the disease continues to infect more people. In the development of such a test, aspects such as sensitivity, specificity, false positive and false negative rates of a diagnostic test have to be meticulously considered. As this is a great undertaking requiring high scientific expertise, laboratory and financial resources, some nations especially low-income settings have had to rely on such support from developed nations to make modifications at a country level so that they reach their healthcare needs ^4^.

The current COVID-19 outbreak has confronted scientists with an unprecedented infectious disease challenge that demands the highest level of sharing of infectious disease-related information as such infectious disease outbreaks are becoming less confined by geographical or climatic boundaries ^5, 6^. Data sharing ensures that other nations use available facts and minimize wastage of time and resources. Another lesson learnt from this pandemic is that nations should also end secrecy in public health decision-making especially amidst suspected disease outbreaks and promote global cooperation ^5^. Globally, governments should be ready to share outbreak information on acquisition, transmission, and treatment as well as make efforts to fight the spreading of ‘fake news’ related to outbreaks. Nations with networks of high-containment biological laboratories must guarantee safety of all agents in their custody to prevent their potential deployment as bioweapons.

This review briefly describes the currently available testing approaches, as well as selected two easy to use point-of-care test assays, including the Xpert ^®^ Xpress SARS-CoV-2 and Abbott ID COVID-19 tests, for diagnosis of novel COVID-19 infection. At the date of this publication, the US Food and Drug Administration (FDA) has granted emergency use authorizations clearance for 32 tests, as seen in Table 1. A routinely updated list of FDA approved tests can be accessed here. 

**Table 1.** Coronavirus disease 2019 (COVID-19) diagnostic tests that have currently received Emergency Use Authorizations (EUA) by the US Food and Drug Administration.

Date EUA issued	Manufacturer	Diagnostic name	Principle action
04/08/2020	DiaCarta, Inc	QuantiVirus SARS-CoV-2 Test kit	qRT-PCR for N, Orf1ab and E genes
04/08/2020	Becton, Dickinson & Company	BD SARS-CoV-2Reagents for BD MAX System	Multiplex qRT-PCR for N1 and N2 regions, and the human RNase P gene
04/07/2020	InBios International, Inc	Smart Detect SARS-CoV-2 rRT-PCR Kit	Multiplex qRT-PCR for N, Orf1ab and E genes
04/06/2020	Gnomegen LLC	Gnomegen COVID-19 RT-Digital PCR Detection Kit	Real-time RT-digital PCR for N1 and N2 regions, and the human RNase P gene
04/03/2020	Co-Diagnostics, Inc.	Logix Smart Coronavirus Disease 2019 (COVID-19) Kit	rRT-PCR for *RdRp* gene
04/03/2020	ScienCell Research Laboratories	ScienCell SARS-CoV-2 Coronavirus Real-time RT-PCR (RT-qPCR) Detection Kit	rRT-PCR for N and Human RPP30 genes
04/03/2020	Luminex Corporation	ARIES SARS-CoV-2 Assay	Multiplex qRT-PCR for ORF1ab, E gene, and N gene
04/02/2020	Becton, Dickinson & Company (BD)	BioGX SARS-CoV-2 Reagents for BD MAX System	Multiplexed qRT-PCR for N1 and RNase P
04/01/2020	Ipsum Diagnostics, LLC	COV-19 IDx assay	Multiplexed qRT-PCR for N and RNase P
04/01/2020	Cellex Inc.	qSARS-CoV-2 IgG/IgM Rapid Test	IgM and IgG antibodies against SARSCoV-2
03/30/2020	QIAGEN GmbH	QIAstat-Dx Respiratory SARS-CoV-2 Panel	Multiplexed qRT-PCR for Orf1b poly gene (Rdrp gene) and E gene
03/30/2020	NeuMoDx Molecular, Inc.	NeuMoDx SARS-CoV-2 Assay	Multiplexed qRT-PCR for Nsp2 gene and N gene
03/27/2020	Luminex Molecular Diagnostics, Inc.	NxTAG CoV Extended Panel Assay	Multiplex qRT-PCR for ORF1ab, and E gene
03/27/2020	Abbott Diagnostics Scarborough, Inc.	ID NOW COVID-19	rRT-PCR for; RdRp gene
03/26/2020	BGI Genomics Co. Ltd	Real-Time Fluorescent RT-PCR Kit for Detecting SARS-2019-nCoV	Multiplex qRT-PCR for N gene, E gene, and RdRp gene
03/25/2020	Avellino Lab USA, Inc.	AvellinoCoV2 test	rRT-PCR for N gene
03/24/2020	PerkinElmer, Inc.	PerkinElmer New Coronavirus Nucleic Acid Detection Kit	Multiplex qRT-PCR for ORF1ab and N genes
03/23/2020	Mesa Biotech Inc.	Accula SARS-Cov-2 Test	rRT-PCR for N gene
03/23/2020	BioFire Defense, LLC	BioFire COVID-19 Test	Nested multiplex real-time RT-PCR for ORF1ab, ORF8
03/20/2020	Cepheid	Xpert Xpress SARS-CoV-2 test	Multiplex qRT-PCR for E and N2 genes
03/20/2020	Primerdesign Ltd.	Primerdesign Ltd COVID-19 genesig Real-Time PCR assay	rRT-PCR for RdRp gene
03/19/2020	GenMark Diagnostics, Inc.	ePlex SARS-CoV-2 Test	rRT-PCR for N gene
03/19/2020	DiaSorin Molecular LLC	Simplexa COVID-19 Direct assay	Multiplex qRT-PCR for ORF1ab and S genes
03/18/2020	Abbott Molecular	Abbott RealTime SARS-CoV-2 assay	Multiplex qRT-PCR for RdRp and N genes
03/17/2020	Quest Diagnostics Infectious Disease, Inc.	Quest SARS-CoV-2 rRT-PCR	rRT-PCR for N gene (N1 and N3 genes)
03/17/2020	Quidel Corporation	Lyra SARS-CoV-2 Assay	rRT-PCR for ORF1ab
03/16/2020	Laboratory Corporation of America (LabCorp)	COVID-19 RT-PCR Test	rRT-PCR for N gene
03/16/2020	Hologic, Inc.	Panther Fusion SARS-CoV-2	rRT-PCR for RdRp gene
03/13/2020	Thermo Fisher Scientific, Inc.	TaqPath COVID-19 Combo Kit	Multiplex qRT-PCR for ORF1ab, N gene, S gene, MS2
03/12/2020	Roche Molecular Systems, Inc. (RMS)	cobas SARS-CoV-2	Multiplex qRT-PCR for ORF-1a and E-gene
02/29/2020	Wadsworth Center, New York State Department of Public Health's (CDC)	New York SARS-CoV-2 Real-time Reverse Transcriptase (RT)-PCR Diagnostic Panel	rRT-PCR for N gene
02/04/2020	Centers for Disease Control and Prevention's (CDC)	CDC 2019-nCoV Real-Time RT-PCR Diagnostic Panel (CDC)	Multiplex qRT-PCR for N1, N2, and RP genes of the virus

## Molecular assays to diagnose COVID-19

The manifestation of the COVID-19 infection is highly nonspecific and presents respiratory symptoms, fever, cough, dyspnoea, and viral pneumonia ^7^. Thus, diagnostic tests specific to this infection are urgently required to confirm suspected cases, screen patients, and conduct virus surveillance. In this scenario, a rapid, robust, and cost-efficient device that can be used anywhere and which does not necessarily require a trained technician to operate ^8^ (i.e. at point-of-care) is crucial and urgently needed for the detection of COVID-19 ^9^. Several assays that detect SARS-CoV-2 have been developed so far, including Rapid IgM-IgG Combined Antibody Test For Coronavirus (RayBiotech Life), 2019-nCoV IgG/IgM Antibody Detection Kit (MyBioSource, Inc.), qSARS-CoV-2 IgG/IgM Rapid Test (Cellex, Inc.), ARIES SARS-CoV-2 Assay (Luminex Corporation), SGTi-flex COVID-19 IgM/IgG (Sugentech, Inc), while still under development is the ultrasensitive, rapid, portable coronavirus SARS-CoV-2 nucleic acid sequence detection system that uses nanobiosensor-based aptamer technology ^10^ are currently under development. These are both *in-house* and commercially available. Some assays detect only the novel form of virus and some may also detect other viral strains (e.g. SARS-CoV) that are genetically similar to SARS-CoV-2 ^11^. More about status of evaluation of SARS-COV-2 molecular diagnostics tests can be found here and here. This includes the viral molecular target utilised in the test, country of manufacturer and its regulatory status.

## Virological culture to diagnose COVID-19

Emerging and re-emerging pathogens are global challenges for public health ^12, 13^. Laboratory biosafety requirements related to COVID-19 virus include the availability of biosafety requirements for viral cultures and further manipulations. The World Health Organisation (WHO) recommends that all procedures must be performed based on risk assessment and only by highly trained personnel with demonstrated capability in strict observance to all relevant protocols at all times ^14^. Non-propagative diagnostic laboratory work, such as viral genome sequencing and nucleic acid amplification tests, must be conducted at containment facilities and procedures equivalent to biosafety level 2 (BSL-2) while propagative work that involves coronavirus culture, isolation, animal inoculation or neutralization assays must be done at a high-biocontainment laboratory with inward directional airflow (minimum BSL-3) ^14^. Viral cultures are not recommended for routine diagnosis and must be carried in a minimum of BSL-3 facility or BSL-4 ^14^. However, SARS-COV-2 virus isolation in cell cultures is critical to obtain isolates for characterization and to support the development of vaccines, therapeutic agents ^15^ and new or better diagnostic tests.

SARS-CoV-2 is isolated and propagated in primary monkey cells and cell lines such as the kidney Vero-E6, LLC-MK2, Human hepatoma cell line Huh7, human airway epithelial cells, and Vero-E6/TMPRSS2 (Transmembrane Serine Protease 2) ^16^. Not all countries or jurisdictions have the facilities to perform virological culture tests for COVID-19. This is due to several reasons such as the required level of technical expertise required for the tests and biosafety requirements. Therefore, in such cases, these regions, e.g. American Samoa, have had to ship clinical samples from suspected individuals to either the US CDC laboratory in Atlanta Georgia ^17^ or WHO reference testing laboratories in France, United Kingdom, China, Japan, Singapore, Australia, Thailand, India, USA, South Africa, Senegal, Russian Federation, Germany, and The Netherlands ^18^. This increases the TAT for the diagnosis even when they are shipped as expedited consignments. 

In addition to virological culture, serological tests are currently under development and these could enable diagnosis of COVID-19 especially in patients for whom acute and convalescent paired samples are available. These are drawn approximately 2 weeks apart to monitor any significant changes in antibody titers of the patients. However, development of these types of tests is currently challenging due to a lack of knowledge about the antibody response elicited from the SARS-CoV-2 infection in humans, including the question concerning the antigenic differences between SARS-CoV-2 and SARS-CoV ^19^. In addition, these serological tests may face a challenge of cross-reactivity with other coronaviruses ^18^. However, the FDA has offered emergency use authorization of the first antibody-based test for COVID-19 that detects antibodies in the one’s blood, rather than for the virus in the nose or throat samples. This test is only done at certified laboratories and even though it takes 15 to 20 minutes to get a result after sample collection, it is not a bedside test ^20^. 

## Point-of-care tests to diagnose COVID-19

Point-of-care testing means that results are delivered to patients in the patient care setting, such as hospitals, urgent care centers and medical emergency rooms, instead of samples being sent to a testing laboratory. Real-time PCR, also known as quantitative PCR, is commonly used in molecular point-of-care testing. It can amplify more than one genomic target and in the case of COVID-19 and these can be *ORF1ab* of coronaviruses occupy about two thirds of their genomes. It encodes the replicase polyprotein and is translated from ORF1a and ORF1b, *E*-gene (envelope protein) and *N*-gene (the nucleocapsid protein) ^21^ and RNA-dependent RNA polymerase (RdRp) that is an essential viral enzyme, which replicates positive-strand RNA viral genome. For more specificity, some molecular diagnostic assays amplify more than one molecular target in the virus to minimize chances of all three targets mutating and hence being missed by the diagnostic test ^21^. During the development of disease testing assays, US manufacturers can apply for emergency clinical use authorization from FDA, and in the European Union manufacturers can obtain "Conformité Européene" which literally means "European Conformity", marking them for *in vitro* diagnostic use.

On March 21, 2020, the US FDA granted emergency use authorization to a rapid, point-of-care diagnostic test designed to detect COVID-19 infection ^22^. This test, Xpert ^®^ Xpress SARS-CoV-2, was developed by Cepheid (Sunnyvale, California, USA) to detect SARS-CoV-2 in approximately 45 minutes, following clinical specimen collection from a nasopharyngeal swab, nasal wash or aspirate. The Xpert ^®^ Xpress SARS-CoV-2 test cartridge is designed to detect nucleic acid from SARS-CoV-2 via real-time PCR ^23^. Xpert ^®^ Xpress SARS-CoV-2 detects two targets, *E* and *N*2 genes ^24^. Furthermore, GeneXpert infinity automated systems do not require users to have special training to perform testing. The Xpert is capable of running 24/7, with many systems already doing so today for other infectious diseases, such as tuberculosis (Xpert ^®^ MTB/RIF) ^25^, *Chlamydia trachomatis* and *Neisseria gonorrhoeae* (Xpert ^®^ CT/NG) ^26^, methicillin-resistant *Staphylococcus aureus* (Xpert ^®^ MRSA) ^27^, Group A Streptococcus (Xpert ^®^ Xpress Strep A) ^28^ and Ebola (Xpert ^®^ Ebola) ^29^.

A second FDA approved test is Abbott ID NOW™ COVID-19, which runs on the Abbott ID NOW™ platform – a lightweight box that can sit in a variety of locations. Positive results can be detected in as little as 5 minutes while negative results take 13 minutes. This test applies molecular technology targeting the COVID-19 *RdRp* gene ^30^. In addition, the above two COVID-19 molecular diagnostic tests are designed for near patient testing in a variety of healthcare environments using a range of sample types such as throat, nasal, nasopharyngeal and oropharyngeal swabs and this facilitates effective patient management.

As the number of confirmed cases of COVID-19 rises rapidly throughout the world, more and more nations continue to require reliable testing. A rapid, inexpensive, and easy-to-use point-of-care diagnostic device integrated with a smartphone could reduce transportation needs, lower the risk of spreading infection, alleviate the strain on the healthcare system, and mitigate the cost of testing for both individuals and governments ^31^. Unfortunately, scaling up production of available COVID-19 diagnostics, as well as personal protective equipment, remain an ongoing challenge in the fight against this disease. A list of other diagnostics under development and testing are listed here ^32^. The WHO prequalification team activated the emergency use listing (EUL) for candidates in vitro diagnostics to detect SARS-CoV-2. There is currently a total of 30 different diagnostic tests at the time of this publication ^33^. Since SARS-CoV-2 diagnostics development and approval are rapidly changing, this information is updated weekly at the WHO portal. As governments around the world are being encouraged by WHO to rapidly test a very large percentage of their populations to fight coronavirus ^34^, these tests will be critical in meeting this demand.

## Whole-genome sequencing for COVID-19

**Figure 1.**
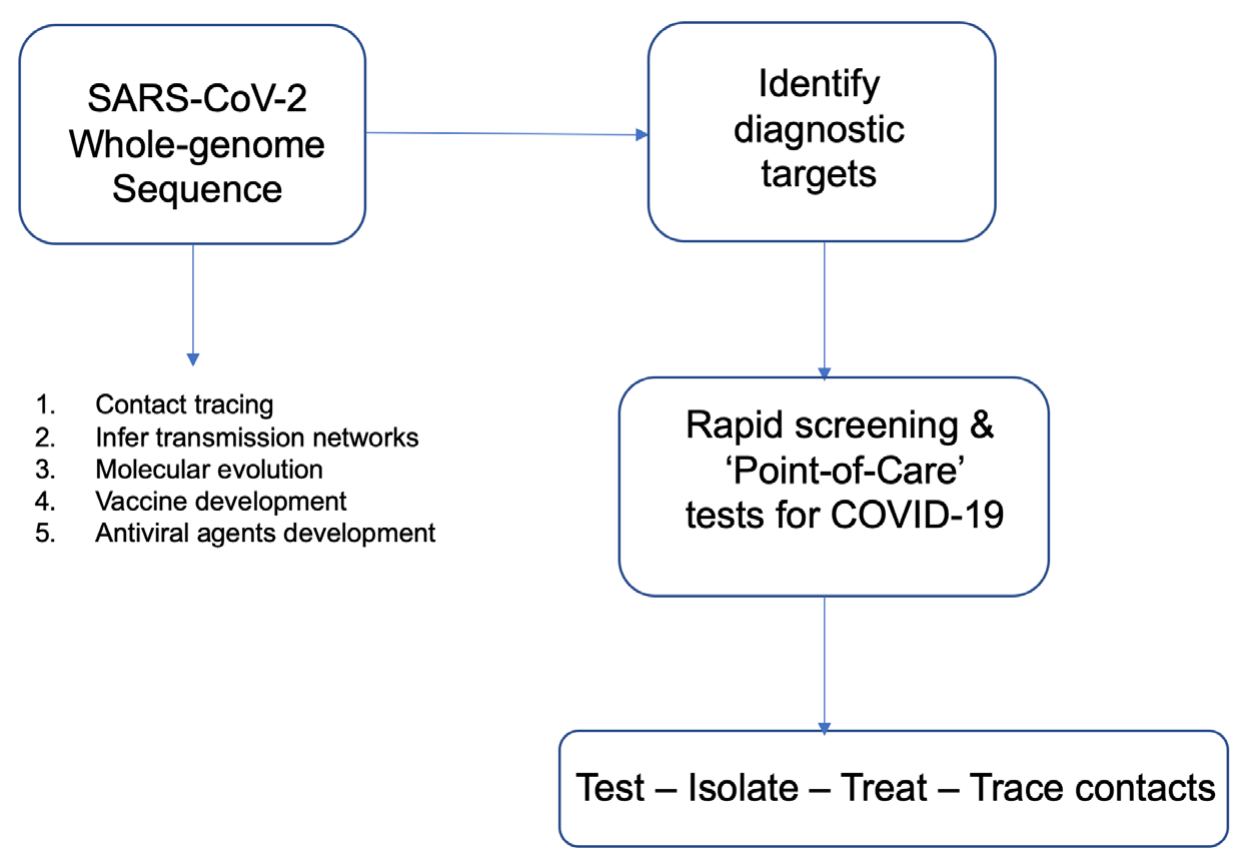
Utility of SARS-CoV-2 whole-genome sequencing.

Following the emergence of SARS-CoV-2, genomic analyses continue to play a key role in the public health response by informing the design of appropriate molecular diagnostics and corroborating epidemiological efforts to trace contacts ^35^. In order to better understand the spread of this pandemic and design better interventions, whole genome sequencing of the virus from a range of clinical presentations of the disease in different parts of the world must occur ( Figure 1). It has been suggested from rapid data sharing using publicly available sequence data platforms that this pandemic was a point-source outbreak ^35, 36^. Therefore, researchers need more data from the whole genomes of SARS-CoV-2 including viral transcriptome ^37^. All these have the potential to reveal further insights into the biology of this emergent pathogen ^35^. Nanopore MinION sequencing technology has been used to sequence SARS-CoV-2 genomes ^38, 39^. Many of these genomes have been successfully uploaded to Global Initiative on Sharing All Influenza Data - GISAID by the WHO reference laboratories globally and Nextstrain. Other public repositories that have a number of these sequences are GenBank and the Sequence Read Archive of the US National Center for Biotechnology.

## The future of COVID-19 rapid point-of-care tests

Currently with more than 2.6 million infected individuals in 185 sovereign states and 183,000 deaths globally ^40^ at the time of writing, the COVID-19 pandemic may see deployment of COVID-19 rapid non-invasive point-of-care tests at civil airports and national borders to screen individuals and avoid imported cases of the infection. This would be similar to mitigation of aircraft hijackings and in-flight sabotage using mandatory civil aviation security procedures, including strict security screening for passengers and baggage at all commercial airports ^41^. Currently, screening for COVID-19 is performed by checking for high fever in individuals coming through airports and border controls using thermal screening guns. A nanosensor diagnostic platform is in development to test for this virus, which will replace thermal screening guns and be more specific. This technology, which will be in the form of a hand-held device, promises to give results specific for this virus within one minute, by detecting the nucleocapsid protein specific for this virus using nanosensor and aptamer technology ^21^.

## Conclusions

To minimise the current COVID-19 pandemic related pressure on health systems globally, especially arising from ramping up testing capacity quickly, readily accessible, cheap, easy to use and interpret point-of-care diagnostic tests with high sensitivity must be developed, produced and distributed widely.

## Data availability

No data is associated with this article.
