# Supplementary material for: Current and emerging diagnostic tests available for the novel COVID-19 global pandemic
Source: AAS Open Res. 2020 Apr 24;3:8. [Version 1] doi: 10.12688/aasopenres.13059.1 (PMC7255909; doi:10.12688/aasopenres.13059.1)
Supplement: Peer review history

# Peer review: Reviewer response for version 1

**DOI:** 10.21956/aasopenres.14154.r27443

**Published:** 2020-05-26

**Authors:** Stephen E. Mshana

**Affiliations:** 1 Microbiology and Immunology department, Weill Bugando School of Medicine, University of Health and Allied Sciences, Mwanza, Tanzania

The letter is well written. It describes the current assays to detect COVID-19. It has clearly outlined the challenges and the need of point of care especially at this time of pandemics. The author needs to add information on sensitivity and specificity of assays for the reader to clearly understand the utility.

Does the article adequately reference differing views and opinions?

Yes

Are all factual statements correct, and are statements and arguments made adequately supported by citations?

Yes

Is the Open Letter written in accessible language?

Yes

Where applicable, are recommendations and next steps explained clearly for others to follow?

Yes

Is the rationale for the Open Letter provided in sufficient detail?

Yes

Reviewer Expertise:

Clinical Microbiology

I confirm that I have read this submission and believe that I have an appropriate level of expertise to confirm that it is of an acceptable scientific standard.

# Peer review: Reviewer response for version 1

**DOI:** 10.21956/aasopenres.14154.r27439

**Published:** 2020-05-06

**Authors:** Anthony Mukwaya

**Affiliations:** 1 Department of Ophthalmology, Institute for Clinical and Experimental Medicine, Faculty of Health Sciences,, Linkoping University, Linköping, Sweden

The novel COVID-19 is thought to have originated from the city of Wuhan in China. This disease is now an ongoing global pandemic that has claimed thousands of lives, and with many more thousands diagnosed with the infection daily. Scientists around the world are working tirelessly to save lives by improving disease diagnosis, patient management strategies, means of containing disease spread, and working towards effective treatments. This calls for concerted action from the different governments, scientists, and the general population to combat this pandemic.

 The review article by Mboowa Gerald titled: ’ Current and emerging diagnostic tests available for the novel COVID-19 global pandemic’, provides good background information on the infection and highlights the challenges faced by scientists, and suggests ways in which to overcome this pandemic. The author clearly and very nicely discusses the current, and emerging diagnostic tests for the novel COVID-19, including molecular and virological assays. The author describes point-of-care tests for disease diagnosis, highlighting; Xpert ^®^ Xpress SARS-CoV-2 and Abbott ID COVID-19 tests, as an example of those that the US-FDA has granted emergency use authorization for diagnosis of the novel COVID-19. Furthermore, the author properly discusses whole-genome sequencing as an important component for disease management, by informing the design of appropriate molecular diagnostic tools, and for a better understanding of the pathophysiology of the disease.

 Taken together, this review article by Mboowa Gerald is detailed, is up to date, and is a timely piece of document which embodies the concerted action required to overcome the current global pandemic.

**Minor comment for the author:**

 In the description of the point-of-care tests for diagnosing COVID-19, the following sentence may be revised for clarity. ‘It can amplify more than one genomic target and in the case of COVID-19 and these can be *ORF1ab* of coronaviruses occupy about two thirds of their genomes’.

Does the article adequately reference differing views and opinions?

Yes

Are all factual statements correct, and are statements and arguments made adequately supported by citations?

Yes

Is the Open Letter written in accessible language?

Yes

Where applicable, are recommendations and next steps explained clearly for others to follow?

Yes

Is the rationale for the Open Letter provided in sufficient detail?

Yes

Reviewer Expertise:

Transcriptomics, angiogenesis, molecular biology, diagnostics, infection, eye diseases

I confirm that I have read this submission and believe that I have an appropriate level of expertise to confirm that it is of an acceptable scientific standard.
